# Incorporation of Waste Glass Powder in the Sustainable Development of Concrete

**DOI:** 10.3390/ma18143223

**Published:** 2025-07-08

**Authors:** Arvindan Sivasuriyan, Eugeniusz Koda

**Affiliations:** Institute of Civil Engineering, Warsaw University of Life Sciences—SGGW, Nowoursynowska 159, 02-776 Warsaw, Poland; eugeniusz_koda@sggw.edu.pl

**Keywords:** waste glass powder, mechanical properties, durability, supplementary cementitious material, sustainable concrete

## Abstract

The steep incline in the rising need for sustainable construction materials has marked the emerging trend of comprehensive research on utilizing waste glass powder (WGP) as a partial substitute for fine aggregates, such as cement, and coarse aggregates in concrete preparation. This review thoroughly examines WGP-incorporated concrete in terms of its mechanical and durability properties. It explores compressive, tensile, and flexural strength, as well as its resistance to freeze–thaw cycles, sulfate attack, and chloride ion penetration. The characteristic microstructure densification, strength development, and durability performance can be attributed to the pozzolanic activity of WGP that forms additional calcium silicate hydrate (C-S-H). The review also highlights the optimal replacement levels of WGP to balance mechanical performance and long-term stability while addressing potential challenges, such as alkali–silica reaction (ASR) and reduced workability at high replacement ratios. By consolidating recent research findings, this study highlights the feasibility of WGP as a sustainable supplementary cementitious material (SCM), promoting eco-friendly construction while mitigating environmental concerns associated with glass waste disposal.

## 1. Introduction

Concrete is a crucial material worldwide due to its strength and versatility in construction. However, producing concrete on a large scale requires a massive amount of cement, which is not environmentally friendly [1]. Manufacturing large quantities of cement contributes to adverse atmospheric changes by releasing carbon dioxide (CO_2_). Concrete is highly versatile and can be designed and molded to meet specific structural requirements. It can withstand heavy loads and perform well in a disaster. It is also less expensive than other global construction materials [2]. Although concrete has many advantages, it also contributes 8% of CO_2_ emissions worldwide due to the large amount of energy required to produce cement, a significant source of binding material in concrete [3]. Early investigations into recycling construction waste revealed the promising potential of various waste-derived materials. For example, Shayan et al. (2004) demonstrated that finely ground glass powder could replace up to 30% of cement while mitigating ASR in concrete [4]. Asokan et al. (2009) explored GRP waste in cement composites, reporting enhanced strength at lower replacement levels and improved long-term performance [5]. Similarly, Ergün et al. (2011) reported that diatomite and waste marble powder could partially replace cement and enhance mechanical properties [6]. Additionally, Federico et al. (2009) reviewed the pozzolanic properties of waste glass, emphasizing size-dependent performance and ASR control strategies [7]. These studies laid the foundational knowledge that supports the current investigation into WGP-based sustainable concrete. Manufacturing cement in industries requires more energy to convert limestone into lime; consequently, CO_2_ is released. Furthermore, cement production is inherently energy-intensive, as it involves the use of fossil fuels to operate at high temperatures [8]. Due to the high carbon footprint released during cement production, researchers are currently focused on developing alternative materials to partially or fully replace cement. This research includes exploring the use of industrial wastes, such as slag, fly ash, and silica fume [9,10]. Additionally, waste glass powder as a partial replacement for cement in concrete has emerged as a significant research topic, given the abundance of glass waste, particularly in European countries [11,12]. WGP is a key substitute material for improving the sustainability of concrete by partially substituting for cement content. WGP is believed to have the ability to interact with calcium hydroxide, yielding additional calcium silicate hydrate, which improves the strength and durability of concrete [13]. Implementing WGP reduces cement usage in the industry and promotes the use of waste glass in the construction sector, thereby contributing to a decrease in CO_2_ emissions [14]. In the pursuit of sustainable construction materials, various recycling strategies have been explored to reduce the environmental impact of concrete. Recent studies highlight that incorporating microfiber into high-toughness recycled aggregate concrete (HTRAC) can significantly enhance stiffness retention and mechanical durability under cyclic loading, making it a strong candidate for structural applications [15]. Similarly, the use of recycled brick powder (RBP) in alkali-activated geopolymer systems has shown potential for low-carbon construction, where optimization of silicate modulus and alkali content has been found to improve both the microstructure and mechanical performance [16]. These advances reflect a broader trend towards valorizing construction and demolition waste, within which waste glass powder (WGP) has emerged as a promising alternative due to its high silica content, pozzolanic reactivity, and abundance in urban waste streams.

**Figure 1:**
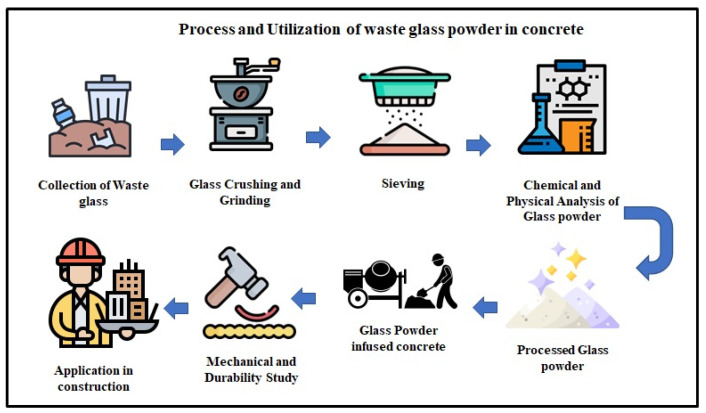
Process flowchart for WGP production and utilization in concrete.

Several investigations have documented the beneficial impacts of WGP in concrete. The strength and durability, as well as the mechanical characteristics, of concrete can be improved by substituting cement with WGP at different ratios. Nevertheless, if the WGP content is excessively increased, difficulties may arise due to a lack of cohesiveness, as well as possible adverse effects from the alkali–silica reaction [17]. With increasing construction work worldwide, finding new sustainable options for traditional materials is crucial. The use of industrial and post-consumer waste in construction work is beginning to be viewed as one approach to alleviating environmental problems and safeguarding natural resources. WGP has emerged prominently among such materials because of its capability to serve as a partial substitute for cement and fine aggregates in concrete. WGP is derived from waste glass cullets, a byproduct of cities and industries. This complements the growing demand for a circular economy, where waste is utilized as a substitute for raw materials [18]. Studies show that WGP’s effectiveness in improving concrete performance greatly depends on its chemical and physical features. With a silica (SiO_2_) content typically exceeding 70%, WGP exhibits pozzolanic characteristics that enable it to react with calcium hydroxide in the cement, producing more calcium silicate hydrate (C-S-H). This contributes to the tremendous increase in concrete’s mechanical strength and durability over time [19]. Additionally, the acceptable size of the WGP increases its surface area, thereby enhancing the reactivity of the WGP to cementitious systems. This improvement in reactivity reduces the likelihood of alkali–silica reaction (ASR), which is typically a problem associated with larger glass particles [20]. Figure 1 illustrates the systematic stages of WGP collection, processing, and incorporation into concrete. It emphasizes processes critical for creating environmentally friendly concrete products, such as glass crushing, particle size classification, chemical testing, and mechanical testing.

WGP achieves sustainability in concrete by reducing cement usage, thereby decreasing CO_2_ emissions. This contributes to solving a significant problem caused by the conventional concrete production process [21]. Furthermore, WGP increases durability and enhances thermal performance, resulting in longer-lasting concrete structures that reduce overall maintenance costs. This improvement reduces the amount of waste materials extracted from the environment and increases the earning potential of construction projects that aim to use eco-friendly materials [22].

Recent improvements in the processing and refinement of WGP have aimed to enhance its performance in concrete applications. Ball milling operations are examples of advanced grinding technologies that enable ultrafine WGP with a particle size of less than 75 μm. Such enhancement furthers the uniformity of the particles and optimizes their pozzolanic reactivity, making high-performance concrete mixtures highly effective [23]. Characterization methods like Scanning Electron Microscopy (SEM) and X-ray Diffraction (XRD) are commonly used to ensure consistent quality and validate the amorphous nature of silica in WGP, which is crucial for its effectiveness [24]. Aside from the mechanical and durability advantages, WGP can potentially mitigate grave issues in waste management. The pace of industrialization and consumerism has led to an upsurge in glass waste produced in metropolitan areas worldwide. Non-biodegradable glass waste is flooding landfills, creating serious environmental problems. Waste glass, which can be recycled into WGP for concrete, serves the purpose of lessening dependence on landfills and conserving limestone and sand, the primary raw materials [25]. Furthermore, the social and economic impacts of WGP integration into construction processes are critical. Utilizing waste materials can lead to cost savings, which is particularly beneficial in regions with limited construction material availability. This helps provide affordable housing and infrastructure, which supports sustainable development targets [26]. Increased collaboration between researchers, policymakers, and industry representatives is necessary to foster the adoption of WGP, which will lead to the promotion of more environmentally friendly construction technologies [27].

The use of WGP in concrete production is economically advantageous, but it poses several challenges. The inconsistency in the quality and composition of glass waste leads to poor performance, so standardized processing techniques are needed. Additionally, the potential for an alkali–silica reaction (ASR) in untreated WGP requires greater emphasis on material processing and quality control. Further research is needed to assess the durability of concrete with WGP composite under various environmental conditions, including freeze–thaw cycles and chemical attacks [28]. Applying waste glass powder in concrete outlines the promise of sustainable construction materials in preventing ecological and economic challenges. Leveraging WGP’s properties while overcoming its limitations can enable the construction industry to reduce its environmental impact while ensuring structural durability and cost effectiveness [29].

### Scope, Bibliometric Insights, and Methodology

**Figure 2:**
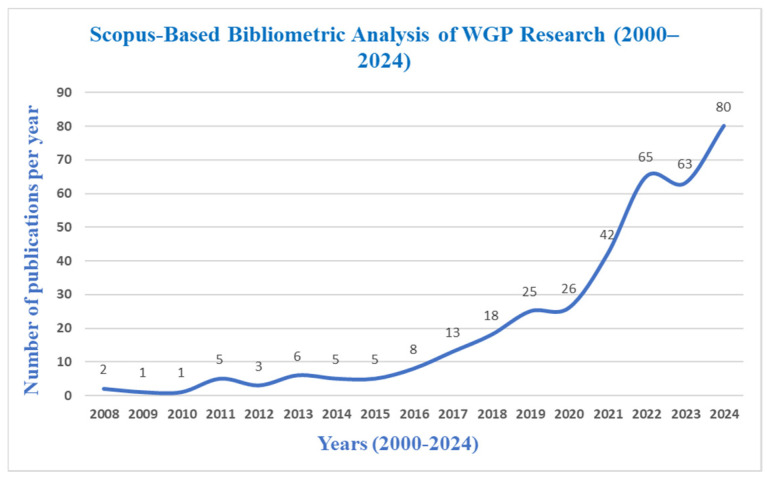
Scopus-based bibliometric analysis of waste glass powder research (2000–2024).

**Figure 3:**
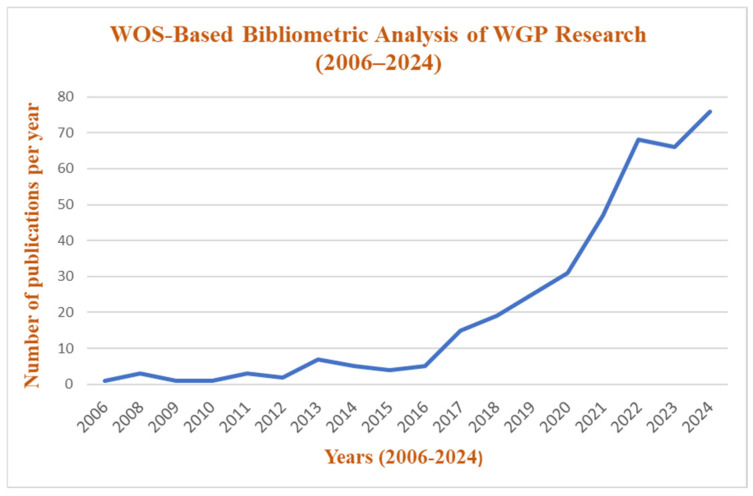
WoS-based bibliometric analysis of waste glass powder research (2006–2024).

The growing concern with the sustainability of construction materials has stimulated research aimed at replacing traditional cementitious materials. WGP has garnered attention due to its pozzolanic properties. This review consolidates existing research on WGP as a partial cement replacement, examining its chemical and physical properties, effects on mechanical and durability characteristics, and overall environmental and economic impact. The primary objective of this study is to assess the feasibility of WGP in concrete production, thus improving sustainability while mitigating waste disposal challenges. By analyzing contributions from academic sources, this review highlights WGP’s role in reducing CO_2_ emissions, optimizing cement consumption, and enhancing concrete’s longevity. Additionally, it identifies critical research gaps, challenges, and future directions for broader implementation in the construction sector. Publication trends were analyzed using bibliometric data from Scopus and Web of Science (WoS). The search queries included “Waste Glass Powder AND Concrete” within the title, abstract, and keywords from 2000 to 2024. Figure 2 illustrates the steady rise in research publications on WGP in concrete, with minimal studies before 2015, followed by a sharp increase from 2018 onwards. The trend shows exponential growth in publications post-2020, indicating a surging interest in sustainable construction materials. The peak in 2024 suggests that WGP has gained significant research attention, reinforcing its role in eco-friendly concrete applications. Likewise, Figure 3 depicts the publication trend of WGP research in WoS from 2006 to 2024. The graph shows minimal research activity until 2015, followed by a gradual increase and a sharp rise from 2018 onwards, similar to the Scopus trend. The rapid surge post-2020 highlights the growing global focus on sustainable cementitious materials, with 2024 marking the highest number of publications, reinforcing the increasing adoption of WGP in concrete research.

A bibliometric assessment of WGP reviews and research over the past two decades based on Scopus, Web of Science, and Google Scholar reveals a steady rise in publications. The primary focus is on WGP’s chemical reactivity, its consequences on mechanical properties, and the prevention of alkali–silica reaction (ASR). Consistent research on sustainable cementitious materials has been predominantly conducted in Europe, North America, and Asia, reflecting widespread global interest in this field. The citation analysis identifies notable studies on particle size optimization, blending strategies, and hybrid cementitious systems that integrate supplementary cementitious materials (SCMs), such as fly ash and silica fume. The effective integration of WGP in concrete aligns with multiple United Nations Sustainable Development Goals (SDGs). It contributes to SDG 9 (Industry, Innovation, and Infrastructure) by progressing sustainable construction materials, SDG 12 (Responsible Consumption and Production) by reducing waste generation, and SDG 13 (Climate Action) by minimizing CO_2_ emissions. Additionally, optimal utilization supports SDG 11 (Sustainable Cities and Communities) by promoting environmentally friendly urban development. Although this review does not represent a full systematic review, a structured approach inspired by the PRISMA (Preferred Reporting Items for Systematic Reviews and Meta-Analyses) guidelines was adopted to enhance transparency and rigor. The relevant literature was identified from Scopus, Web of Science (WoS), and Google Scholar using the keywords “Waste Glass Powder AND Concrete” across the title, abstract, and keywords from 2000 to 2024. Articles were screened based on their relevance to WGP use in cementitious systems, with a focus on mechanical performance, durability, sustainability, and environmental impact. Non-English publications and duplicates were excluded. This strategy ensured a comprehensive yet focused literature base, supporting the thematic synthesis and trend analysis presented in this review.

This review highlights the need for standardization, large-scale implementation, and cross-disciplinary collaboration to establish WGP as a suitable and sustainable supplementary cementitious material. Although several reviews have addressed the use of WGP in cementitious materials, most focus narrowly on either mechanical performance or pozzolanic reactivity, often lacking an integrated discussion across multiple performance aspects. Furthermore, few studies provide a systematic bibliometric perspective or align their findings with current sustainability agendas, such as the SDGs. This review addresses these gaps by offering a comprehensive synthesis of WGP’s influence on chemical, mechanical, and durability performance while incorporating bibliometric trends and sustainability linkages. The novelty of this review lies in its cross-disciplinary approach, which connects material science, environmental impact, and construction policy perspectives to guide future research and implementation.

## 2. Properties of Waste Glass Powder

WGP has gained considerable interest as a supplementary cementitious material (SCM) owing to its essential physical and chemical properties. Its elevated pozzolanic reactivity, high microstructural refinement, and excellent strength in cementitious materials result from its fine particle size and substantial silica content. To realize WGP’s full potential in sustainable concrete production, a comprehensive understanding of its chemical composition, physical properties, and processing methods is necessary.

### 2.1. Chemical and Physical Properties

The physical and chemical properties of WGP have a significant impact on its reactivity, longevity, and usefulness as a supplementary cementitious material (SCM). Knowing its composition, particle size distribution, and surface characteristics enables the optimization of pozzolanic activity, microstructure refinement, and overall compatibility within cementitious systems. In an ultra-high-performance concrete (UHPC) analysis containing WGP, Abellan-Garcia et al. [30] studied 28MRG and 7MRG, which were jet-milled to 28 and 7 microns, respectively. XRD indicated an amorphous nature, and chemical analysis revealed the presence of high SiO_2_, ranging from 72 to 75%, and alkali (Na_2_O > 10%). SEM imaging also revealed smooth angular particles, which contributed to enhancing pozzolanic activity and microstructural refinement and reducing porosity. Singh and Mohanty [31] observed ~70.2% Si and 11.4% alkali in WGP for alkali-activated concrete (AAC) with FA and GGBS. Physical characterization revealed a median particle size of 11.74 microns and a specific surface area of 6230 cm^2^/g, indicating high reactivity. SEM revealed irregular and angular particles, which enhanced the formation of C-S-H and C-(N)-A-S-H gels, refined the microstructure, and improved the durability of AAC.

**Figure 4:**
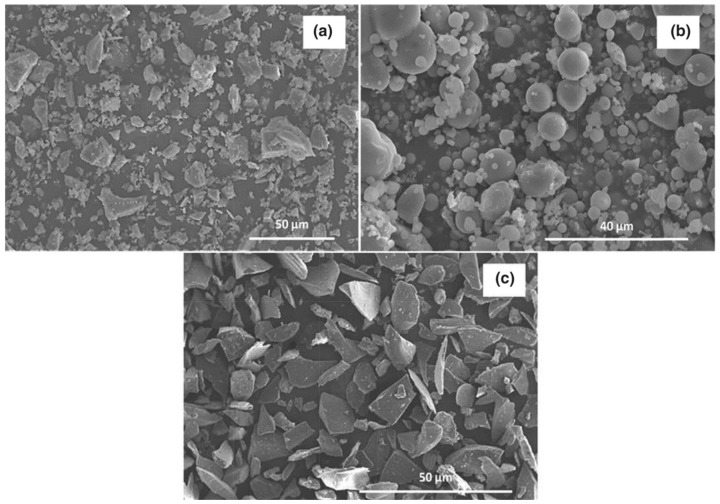
SEM micrographs of raw binders showing (**a**) GP cement, (**b**) FA, and (**c**) coarse PGP. (Reproduced from Mahmood et al. (2022) [34] under CC BY 4.0 License).

Yavuz et al. [32] investigated WGP in porous concrete (PC), confirming that SiO_2_ accounts for ~70.1%, making it a reactive pozzolan. Physical analysis showed a particle size of 40 µm and a surface area of 3416 cm^2^/g, reflecting high reactivity. XRD confirmed the amorphous phase, while SEM revealed a smooth yet irregular morphology, which enhances microstructure, compactness, and durability, particularly at later curing stages. Chen et al. [33] studied WGP for alkali–silica reaction (ASR) mitigation in tunnel waste rock slag-based concrete, confirming SiO_2_ ~77.32% and Na_2_O ~10.27%. Physical tests showed particle sizes from 2.5 µm to 150 µm, where particles < 75 µm inhibited ASR, while larger particles accelerated it. SEM confirmed that finer WGP promoted pozzolanic activity, consuming excess alkalis, reducing ASR gel formation, and enhancing durability. Figure 4 highlights the morphological characteristics of the SEM micrographs of raw binders, including GP cement, fly ash (FA), and coarse pulverized glass powder (PGP). The microstructural analysis provides insights into the particle shape and texture, which influence the pozzolanic reactivity and performance of these materials in concrete applications. While particle morphology (shape and texture) contributes to pozzolanic activity, particle size distribution is a more critical factor. Mahmood et al. [34] demonstrated that finely ground pulverized glass powder (PGP) with a median particle size (D50) of 19.3 µm exhibited significantly greater strength performance than coarse PGP or fly ash, highlighting the importance of ultrafine grinding for enhanced reactivity.

Yuan et al. [35] assessed WGP with eggshell particles (ESP) in high-strength eco-friendly concrete (ESGPC), confirming SiO_2_ ~73%, Na_2_O ~13%, and CaO ~11.3%. The median particle size of 16.75 µm improved pozzolanic reactivity and the filler effect. SEM revealed prismatic particles, which improved void filling and pore distribution, thereby increasing compactness, durability, and mechanical performance. Yoo et al. [36] investigated waste liquid crystal display glass powder (LCDGP) in ultra-high-performance concrete (UHPC), reporting SiO_2_ at ~67.3% and Al_2_O_3_ at ~18.9%, which confirms its pozzolanic reactivity. XRD analysis verified its amorphous nature, while SEM displayed angular particles (~10.4 µm size), similar to cement. LCDGP refined the interfacial transition zone (ITZ), increased shear resistance, and improved mechanical and durability properties, making it a sustainable SCM.

### 2.2. Preparation and Processing Methods

The processing of WGP effectively impacts its chemical reactivity and mechanical performance in cementitious applications. Yasouj and Ghaderi [37] examined WGP as a replacement for cement in green concrete, demonstrating that WGP particles < 75 µm exhibited high pozzolanic reactivity, improving the microstructure and mechanical properties. Chemical analysis confirmed SiO_2_ ~72%, supporting additional C-S-H gel formation. SEM demonstrated that WGP minimized voids and cracks, enhancing its density, durability, and permeability resistance, making it a feasible SCM for sustainable construction. Ramakrishnan et al. [38] assessed WGP combined with GGBS as a partial replacement for cement, confirming a SiO_2_ content of ~72%. Fine particles of WGP (<75 µm) ensured high pozzolanic activity and ASR prevention. The physical experimentation yielded a specific gravity of 2.89 and a fineness of 390 m^2^/kg, comparable to that of cement. At the range of 15% replacement, WGP minimized voids, densified the cement matrix, and enhanced the microstructure, confirming its suitability as an SCM for sustainable concrete. Ruan et al. [39] assessed WGP with recycled waste concrete powder (RWCP) in foam ceramics, demonstrating SiO_2_ content of ~68.41% and Na_2_O content of ~15.7%, which improves pozzolanic reactivity at high temperatures. The median particle size of 14.6 µm enhanced reactivity and uniform blending. XRD confirmed the amorphous property, while SEM revealed smooth surfaces and irregular morphology, which influenced the pore structure, crystalline phase formation (mullite, pyroxene, and wollastonite), and microstructure improvement. Arslan et al. [40] evaluated waste glass aggregate (WGA) as a partial replacement for quartz sand in reactive powder concrete (RPC). Chemical tests confirmed SiO_2_ ~70.2% and Na_2_O ~12.0%, while physical analysis showed a specific gravity of 2.60 g/cm^3^, enhancing packing density and reducing porosity by 47.5% at optimal replacement. SEM confirmed that up to 30% of WGA replacement improved the microstructure and ITZ, but higher levels caused poor compaction and air entrapment due to WGA’s lightweight nature. Optimized use enhanced RPC durability and structural integrity.

Mo et al. [41] investigated ultrafine WGP in crumb rubber concrete (CRC), showing SiO_2_ ~57.42% with particle sizes from 0.2 to 10 µm (mean diameter: 2.5 µm). SEM revealed smooth, fine WGP particles, which improved packing density, reduced voids, and refined the ITZ. These modifications reduced porosity, improved mechanical performance, and decreased chloride ion permeability, thereby supporting WGP’s viability as a sustainable SCM. Tian et al. [42] examined WGP and recycled waste concrete powder (RWCP) in foam ceramics, confirming SiO_2_ at ~68.41% and Na_2_O at ~15.7%, which reduced the sintering temperature. Physical tests revealed a median WGP particle size of 14.6 µm, indicating suitability for blending. XRD confirmed amorphous silica dominance, improving reactivity, pore structure, compressive strength, porosity, and water absorption in foam ceramics. Madandoust and Ghavidel [43] explored the use of WGP combined with rice husk ash (RHA) as a cement replacement, reporting SiO_2_ at ~73.1% and Na_2_O + K_2_O at ~11.1%, which supports the pozzolanic behavior. The fineness of 9768 cm^2^/g ensured high reactivity, mitigating ASR, improving pore structure, and enhancing the compactness of the concrete. A 10% WGP and 5% RHA blend boosted the strength activity index and microstructure, making WGP an effective SCM. Su and Xu [44] investigated WGP with RHA as supplementary cementitious materials (SCM), confirming SiO_2_ content of ~70%. Physical analysis revealed a particle size of 40 µm, which improved the fluidity and microstructure by reducing voids and cracks. At 20% WGP replacement, significant enhancements in pozzolanic reactivity, compressive strength, and durability were observed, highlighting the synergy between WGP and RHA in reducing water absorption and improving performance.

Sathiparan and Subramaniam [45] reviewed WGP as a supplementary cementitious material (SCM) in pervious concrete, confirming SiO_2_ ~70–75% and particle size < 75 µm, enhancing packing density and reactivity. At 10–15% replacement, WGP improved compressive strength and durability due to C-S-H formation, but higher ratios reduced strength and increased freeze–thaw susceptibility. The study emphasized WGP’s sustainability benefits in reducing cement consumption and environmental impact. Hwang and Moreno Cortés [46] analyzed milled WGP and coal fly ash (FA) as SCMs in mortar and pervious concrete, reporting SiO_2_ of ~59.47%, a specific surface area of 358 m^2^/kg, and a median particle size of 8 µm (ball-milled). WGP’s fine particles improved compactness, reduced voids, and increased compressive strength and durability. The optimal 7.3% WGP replacement significantly enhanced the performance of mortar and previous concrete, demonstrating its sustainability potential. Yan et al. [47] investigated WGP in green ultra-high-performance concrete (UHPC), confirming SiO_2_ ~65.77%, CaO ~13.23%, Na_2_O ~9.50%, and a density of 2.69 g/cm^3^. SEM revealed smooth and irregular particles, which improved packing density and reduced porosity. At a 20% replacement rate, WGP increased compressive strength by 8.6% at 28 days; however, higher replacement rates led to cement dilution. Saribiyik et al. [48] investigated WGP as a replacement for quartz aggregate in polymer concrete, reporting a SiO_2_ content of ~72% and a fine particle size (<1 mm), which improved compactness and filling capacity. WGP replacement (10–47%) increased compressive strength (up to 29%). Flexural strength (up to 78%) is achieved while enhancing workability, highlighting the sustainability and cost-effectiveness of polymer concrete, as shown in Table 1, which provides an overview of significant studies investigating the chemical and physical characteristics of WGP in cement-based materials.

## 3. Mechanical Properties of Concrete with WGP

Using waste glass powder in concrete has garnered considerable attention due to its ability to improve mechanical properties while progressing sustainability objectives. As a replacement cementitious material, WGP significantly improves compressive, tensile, and flexural strengths by promoting pozzolanic activity and transforming the microstructural framework. Its fine particle size and elevated silica content enhance the development of calcium silicate hydrate (C-S-H) gels, contributing to a denser cementitious matrix and improved durability. Moreover, WGP enhances long-term durability by successfully mitigating the alkali–silica reaction (ASR), positioning it as a possible alternative to conventional cementitious components.

Safi et al. [49] studied the effect of using waste glass powder (WGP) as a partial cement substitute on mechanical performance. Their results show that replacing cement with WGP at an optimal 10% to 20% enhances compressive strength. In particular, 17.5% replacement resulted in a significant improvement of 6.07% in compressive strength and 6.85% in flexural strength over control mixtures after 28 days of curing. Similarly, Zeybek et al. [50] achieved improved bond strength in concrete when using WGP at a 20% replacement level.

### 3.1. Compressive Strength

Concrete’s strength is one of the most important criteria for its mechanical performance, mainly when WGP is used as a substitute material. There have been claims that the WGP aids compressive strength due to its pozzolanic activity and particle packing effect. Simultaneously, excessive substitution can result in a dilution of the cementitious components, leading to a deterioration in strength. Lai and Chen [51] noted a 56.8% increase in strength from the combination of 15% glass micro-powder (GMP) and 30% construction waste due to cavity filling, with C-S-H and C-A-H being the primary causative structures. Additionally, Khan et al. [52] observed that the highest compressive strength was recorded at 10% WGP replacement for cement and 15% for sand, with a decline in strength noted beyond this point due to reduced pozzolanic activity and increased porosity.

Concerning ultra-high-performance concrete (UHPC), Li et al. [53] showed that compressive strength improved by 5.61% when 20% WGP and oyster shell powder (OSP) were used. However, further application of these materials resulted in a weakening effect due to the formation of deficient hydration products. Miao et al. [54] applied machine learning to optimize WGP-based concrete mix designs. They found 15% WGP replacement to be the most effective level while minimizing delayed hydration effects for compressive strength enhancement. The role of WGP in cementitious systems was further explored in the context of seashell powder calcined sludge cement (SCSC) by Gong et al. [55]. Their study showed that at 5% WGP replacement, the strength increased by 25.26%. However, at higher replacement levels, the strength decreased due to excessive dilution of cementitious constituents beyond 15%. Similarly, Song et al. [56] investigated the use of WGP with recycled aggregates. They found that early-age compressive strength is initially lowered but increases significantly after 90 days due to the formation of secondary C-S-H gel. In conventional concrete applications, Safarizki et al. [57] observed that replacing sand with WGP at a rate of 15% resulted in an optimal compressive strength of 22.8 MPa. Any further increases resulted in a reduction in strength because of poor bonding of the aggregates. Rahman and Uddin [58] also noted stronger plain concrete at 20% WGP cement replacement, although a 15% strength reduction was experienced at 30% replacement due to the dominant bonding failure. Lopez and El-Fata [59] examined the use of WGP to increase the sustainability of concrete. They reported that the compressive strength of grade 20 concrete was up to 10 MPa, but excessively replacing concrete leads to poor interfacial bonding. Lastly, Mohammadi Golafshani and Kashani [60] used machine learning on 830 records to estimate the performance of WGP concrete. They verified that fine particles of WGP increase strength, but larger particles reduce the bonds of the aggregate paste.

### 3.2. Tensile and Flexural Strength Analyses

The evaluation of concrete incorporating WGP begins by examining its mechanical properties, which include tensile and flexural strength. Multiple researchers have studied the impact of WGP, either solely or in conjunction with other constituents, on the progress of these attributes while practicing eco-friendly procedures during concrete processing. Hitesh Kumar et al. [61] studied the interaction of WGP with Recycled Steel Fibers (RSF) on the tensile and flexural strength of concrete. The optimum combination was observed to be 9% WGP and 1% RSF due to enhanced crack bridging and energy absorption. Beyond 9% WGP, the strength progressively lowered due to a high volume of voids and dilution of the cementitious phase.

Orouji and Najaf [62] added WGP incorporating glass-fiber-reinforced polymer (GFRP) rebars and polypropylene (PP) fibers. While a 10% WGP substitution negatively impacted flexural strength due to increased brittleness, adding 1.0% polypropylene fiber significantly enhanced crack resistance and ductility. Orouji et al. [63] also reported that 25% WGP, in combination with 1.5% PP fibers, gave the highest flexural strength and toughness. The study reported a fourfold increase in toughness and a 13-point increase in energy absorption. However, the excessive content of PP fibers led to adverse effects on workability. Mustafa et al. [64] evaluated recycled GP and steel fiber (SF) in reinforced concrete slabs, finding that 10% GP replacement increased failure load by 8.8%, while 0.5% and 1% SF improved flexural strength by 20.6% and 38.2%, respectively. Table 2 highlights the impact of WGP incorporation on the mechanical strength performance of concrete.

Shi-Yi He et al. [65] examined waste glass aggregate in concrete-filled steel tubes (CFST). The research demonstrated that 40% replacement of glass aggregate resulted in a 6% reduction in flexural strength, while total replacement caused a 33% reduction. However, integrating perforated rib shear connectors (PBL) and steel reinforcements enhanced flexural stiffness and ductility. Consequently, Ravikanth et al. [66] utilized a supervised data-driven approach to predict the tensile and flexural strength of WGP concrete, confirming that optimized WGP replacement improved stability but excessive content led to brittleness. Salas et al. [67] studied WGP as an acceptable aggregate substitute, showing a 14.41% increase in tensile strength at a 10% replacement level. However, beyond 20%, microvoids reduced the tensile performance.

Dey et al. [68] analyzed geopolymer concrete with WGP and gold mine tailings (MT), finding that the splitting tensile strength improved due to enhanced matrix densification, although the flexural gains were limited. Lastly, Li et al. [69] conducted uniaxial tensile tests on WGP concrete, revealing that higher loading rates improved the ultimate tensile strength, while energy absorption decreased with increased WGP content, which affected the resistance to dynamic loads.

## 4. Durability Analysis

Durability plays a significant role in determining the prolonged performance of concrete, specifically under adverse environmental conditions. The integration of WGP has demonstrated the potential to improve mechanical performance and durability. However, an in-depth evaluation must outline its resistance to critical durability challenges. This review assesses three major durability issues that significantly affect the concrete’s structural integrity and service life: freeze–thaw cycles, sulfate attack, and chloride ion penetration.

In cold regions, freeze–thaw cycles can lead to severe degradation of concrete as water enters its pores, freezes, and expands, resulting in microcracks, surface scaling, and loss of strength. As WGP contributes to the refinement of pores and reduces permeability, its capabilities in improving resistance to frost warrant further investigation. Conducting a detailed investigation of WGP’s pozzolanic activity and the restrictions it imposes on calcium hydroxide availability is sure to enhance our understanding of how it improves sulfate resistance [70]. Moreover, the penetration of chloride ions poses a significant threat to durability in marine environments and regions subjected to de-icing salts, as it accelerates the corrosion of steel reinforcement and deteriorates the structural integrity. Evaluating the effectiveness of restricting chloride penetration is essential for enhancing the durability of reinforced concrete, as WGP aids in matrix densification and reduces chloride permeability [71].

### 4.1. Freeze–Thaw Cycles

In colder regions, concrete buildings are susceptible to damage from freezing and thawing (FT), a process in which the expansion and contraction of water within the pores lead to destruction over time. Researchers have investigated the use of WGP as a sustainable option to enhance frost resistance and mitigate damage.

Li et al. [72] investigated the freeze–thaw resistance of earlier proposed concrete composites with WGP. Their findings showed that substituting 10% cement for WGP results in an enhanced dynamic elastic modulus and durability that is 60% greater than that of ASTM C666 after 300 freeze–thaw cycles. Abendeh and AbuSalem [73] studied the use of recycled glass powder (RGP) as a partial replacement for cement and its effects on compressive strength retention at lower RGP replacement levels. However, higher replacement proportions led to microcracking and increased permeability under cyclic freezing and thawing conditions.

He et al. [74] studied the frost resistance characteristics of concrete blended with waste rubber and WGP. Their research results illustrated that the use of both materials reduced mass loss while increasing residual strength after 200 freeze–thaw cycles, demonstrating the synergistic effects of passive elastic rubber particles and cementitious WGP. Lee et al. [75] studied the durability of concrete prisms incorporated with WGP and waste glass sludge. WGP assisted in pore refinement, along with hydration reprocessing, and ensured adequate surface scaling during freeze–thaw cycles. Kim et al. [76] made preliminary efforts to evaluate the durability of concrete containing waste glass, limiting sludge after subjecting the concrete to freeze–thaw cycles and de-icing salt attacks. Their findings indicated that finely ground waste glass sludge improved the concrete’s freeze–thaw resistance. Still, high proportions resulted in surface deterioration when exposed to de-icing salts, illustrating the need for optimized replacement ratios.

### 4.2. Sulfate Attack

Sulfate introduced in concrete causes matrix deterioration and structural damage due to the generation of expansive ettringite. Recent studies have evaluated the potential resistance to sulfate from various glass waste constituents, including LCD glass powder, recycled glass aggregate from used beverage bottles, and crushed waste glass. These materials help to decrease the permeability and the negative impacts of sulfate attack by enhancing the porosity of concrete. Kim and Hong [77] investigated the sulfate resistance of reinforced concrete flumes containing LCD waste glass powder. Their research studied replacement proportions of 10% and 20%. It was noted that the sulfate’s durability was increased due to the fine particle size of LCD waste glass powder (5 μm). This was due to low porosity and high compressive strength, especially for construction materials that had been exposed to high concentrations and long durations of sodium sulfate (Na_2_SO_4_) and magnesium sulfate (MgSO_4_) solutions. Cao et al. [78] investigated the effect of glass powder on external sulfate attack (ESA) in cementitious materials, focusing on the sulfate resistance and alkalinity of the pore solution. These findings showed that the formation of ettringite and gypsum was diminished. Their consequences indicated that the development of gypsum and ettringite was minimized by incorporating glass powder, which effectively controls sulfate-induced expansion and deterioration.

Mostofinejad et al. [79] initially analyzed the strength of concrete containing shredded waste glass and coarse and fine recycled aggregates when exposed to magnesium sulfate. They found that incorporating milled glass waste accelerated the deterioration of cement mortar in a sulfate environment and under pH control; however, excessive glass content led to microstructural weaknesses resulting from prolonged exposure to sulfate. Siad et al. [80] studied the behavior of mortar infused with glass powder when exposed to sulfuric acid instead of sulfate. Their results suggested that the inclusion of glass powder contributed to a more active pozzolanic reaction in matrix densification and slowed the rate of destruction from acid sulfate reactions. Tanwar et al. [81] investigated the use of waste beverage glass (WBG) as a substitute for fine aggregates in concretes containing ground granulated blast-furnace slag (GGBS) in sulfate-rich environments. Their results showed that replacing WBG in the 5–10% range enhanced resistance to sulfates by improving particle packing. Microcracking, believed to occur due to the angular morphology of the waste glass particles, was found to occur at replacement rates over 15%, as cited in the reference. Similarly, Jain et al. [82] analyzed the durability of concrete containing a blend of granite and waste glass powder, as well as its resistance to sulfate attack. Their results indicated that cement with high tricalcium aluminate (C3A) and 20% waste glass powder (WGP) replacement exhibited excellent resistance to sulfate deterioration. Kubatova et al. [83] studied the resistance of geopolymer binders made with fluorescent lamp glass waste powder to sulfate and sulfuric acid. They concluded that waste glass lightened the matrix’s destruction because the fractured geopolymer binders built a stable aluminosilicate network. The binders were less resistant to sulfate deterioration than ordinary Portland cement, but they were more resistant to sulfate degradation caused by the cement.

### 4.3. Chloride Ion Penetration

Chloride ion penetration poses a significant durability challenge in reinforced concrete structures, accelerating steel corrosion and reducing service life. Environmental and Economic Impact: The issue of chloride ion penetration is one of the most significant challenges to the durability of reinforced concrete structures over time, as it leads to steel corrosion and reduces the structure’s lifespan. Scientists have investigated the potential of utilizing waste glass powder and its derivatives as a beneficial and environmentally friendly solution to enhance matrix densification by refining the pore structure and increasing the resistance of concrete to chloride penetration. Peng et al. [84] looked at the corrosion resistance of recycled aggregate concrete incorporating waste glass powder. Their results showed that WGP enhances the durability of reinforced concrete structures by increasing the density of the matrix, lowering permeability, and reducing the ingress of chloride ions, which leads to chloride-induced corrosion. Similarly, Du and Tan [85] noted that replacing cement with WGP results in lower levels of chloride ion penetration. They attributed this improvement to pozzolanic activity, which increased chemical resistance by reducing porosity and mitigating chloride-induced deterioration. Dong et al. [86] investigated the use of recycled waste glass (RWG) powder, sand, and cullet in producing cementitious and geopolymer concretes. Their research showed that larger quantities of glass resulted in lower chloride ion ingress due to greater secondary hydration and a refined pore structure. Paul et al. [87] investigated the feasibility of using crushed waste glass (CWG) as a substitute for coarse aggregate in green concrete. Their results showed that it enhanced the pore structure, thereby increasing durability by reducing chloride ion permeability and providing an environmentally friendly approach to counteracting chloride-induced corrosion of reinforced concrete structures. Ahmed [88] evaluated the incorporation of glass waste nanopowder (GWNP) into magnetic concrete and found that nano-scale GWNP-refined powders decreased chloride diffusion coefficients. With this synergistic effect, GWNP-modified concrete becomes a candidate material for structural components that require high performance and low corrosion resistance.

## 5. Environmental and Economic Impact

**Figure 5:**
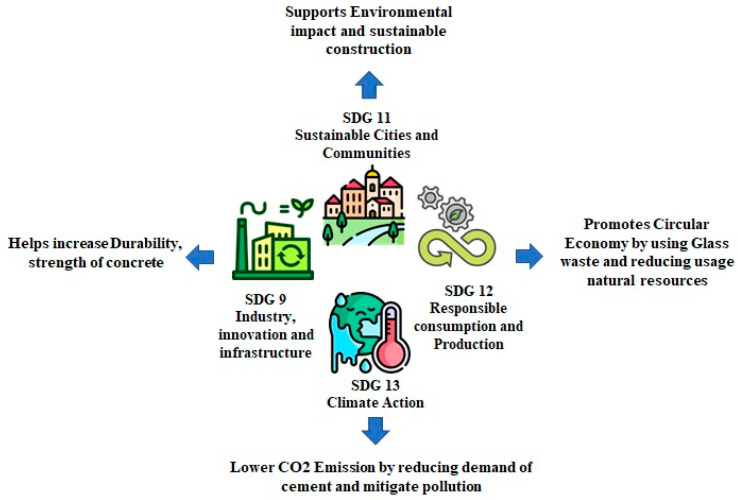
Sustainability impact of waste glass powder utilization in concrete, highlighting its contribution to SDG 9, SDG 11, SDG 12, and SDG 13.

The integration of WGP into concrete represents a significant advancement in developing sustainable and economically viable construction materials, aligning with the principles of a circular economy, as illustrated in Figure 5. Given that the cement industry contributes nearly 8% of global CO_2_ emissions, replacing cement with WGP provides viable strategies to minimize environmental impact while preserving natural resources [89]. Furthermore, applying WGP in concrete contributes to effective waste management by reusing significant quantities of post-consumer glass, thereby reducing landfill accumulation while enhancing the mechanical properties and longevity of concrete structures. The economic feasibility of integrating WGP arises from its reduction in reliance on raw materials, potential reductions in overall energy consumption during cement production processes, and cost effectiveness. Considering the dual impact of WGP-based concrete on ecological and economic feasibility, this section examines the reduction of CO_2_ emissions, advancements in waste management, and improvements in resource efficiency [90].

a. Reduction in CO_2_ emissions.

The cement industry accounts for approximately 8% of global CO_2_ emissions, a significant contributor to climate change. The cement industry is known to contribute around 8% of global CO_2_ emissions, which is a considerable concern for global warming. Using WGP as a partial substitute in cement helps lower emissions without compromising the concrete’s structural properties. Ibrahim [91] explored the effectiveness of WGP as a partial replacement for cement. He found that WGP can substitute for cement, resulting in one ton of CO_2_ emissions being saved for every ton of cement replaced with WGP. Olofinnade et al. [92] also studied the effects of ground waste glass powder (GWGP) on carbon dioxide emissions in concrete. Their results confirmed that the use of GWGP in cement as a substitute reduces energy and emissions in the process, thereby contributing to sustainable construction.

**Figure 6:**
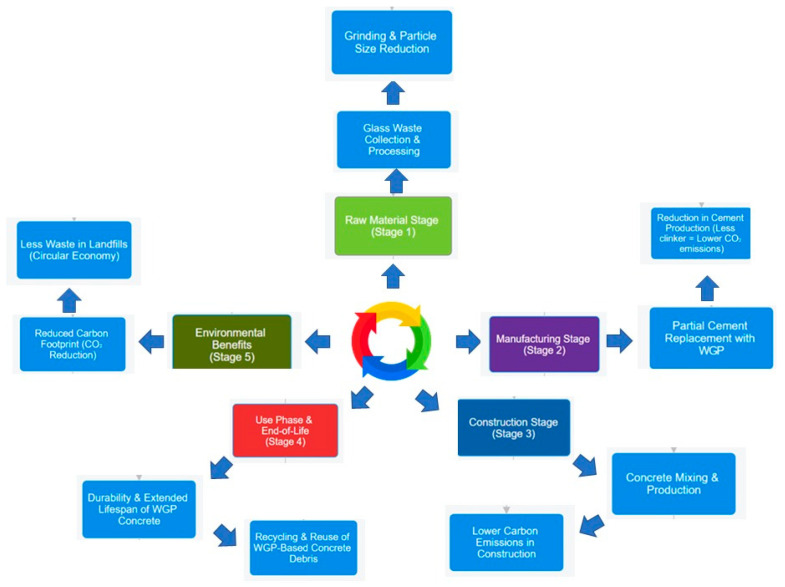
WGP life cycle assessment (LCA) of concrete shows that raw materials play a crucial role in reducing CO_2_ emissions during processing, development, and construction and in maintaining long-term stability.

Aliabdo et al. [93] demonstrated reduced carbon emissions during cement production by replacing cement with WGP. Their study established that for every ton of Portland cement replaced with WGP, nearly one ton of carbon emissions is captured, demonstrating its potential as a replacement for Portland cement. Kalakada et al. [94] investigated the microplastic size distribution of WGP in cementitious composites and found that some fine glass particles have a positive effect on pozzolanic activity. The resulting pozzolanic activity is combined with an increase in the cement’s efficiency, resulting in a more significant reduction in carbon dioxide emissions. Tamanna and Tuladhar [95] studied the effectiveness of crushed recycled clear glass powder in concrete for its long-term performance. Their research showed that the use of concrete contributes significantly to reducing both embodied carbon and landfill waste compared to other materials used in the construction industry. The complete life cycle assessment of WGP in concrete is presented in Figure 6, which indicates the reduction in carbon dioxide emissions associated with all phases, from materials processing to the sustainability phase. While WGP offers significant environmental advantages by diverting glass waste from landfills and reducing cement consumption, it is essential to acknowledge potential trade-offs. The grinding and processing of waste glass into ultrafine powder require substantial energy input, particularly when aiming for particle sizes below 75 µm to enhance pozzolanic activity. This process may offset some of the environmental benefits, particularly in regions with high levels of fossil-fuel-based electricity. Additionally, resource extraction (e.g., water for cooling during grinding) and dust management may impose ecological burdens. Hence, a complete life cycle assessment (LCA) is essential to quantify net sustainability gains and ensure the process remains eco-efficient.

b. Waste Management Benefits.

The glass-containing wastes currently discarded in landfills pose a threat to the environment, as they do not decompose, and recycling is often impossible in many places. Replacing cement with waste glass powders facilitates effective partial diversion of glass waste from landfills and enhances resource utilization in construction as a cementitious material. Jiang et al. [96] gave a thorough and comprehensive review of the roles of WGP in cement-based materials. They pointed out that it yields the dual advantages of decreasing waste in landfills and augmenting the cementitious characteristics. These results suggest the potential use of WGP as a candidate for achieving sustainability in the construction industry.

Agboola et al. [97] investigated the role of WGP in manufacturing concrete as a pozzolana. It was demonstrated that substituting WGP into concrete cycles significantly reduces waste sent to landfills while enhancing the strength properties of the cementitious materials, thereby making it a more environmentally friendly option than conventional waste treatment approaches. Qin et al. [98] analyzed the consequences of incorporating WGP into cement-based materials and its role in environmentally sustainable construction and waste minimization. Their results verified that WGP in fine particles has a higher level of reactive silicates. In addition, research underlined that including WGP in concrete reduces landfill waste and reduces dependence on raw material extraction for cement production, supporting changes in the construction sector towards the circular economy. Hamada et al. [99] reviewed the consequences of adding recycled waste glass to high-performance concrete, highlighting the potential for reducing waste disposal costs and mitigating pollution. The research emphasized that effective waste glass utilization in cement and aggregate forms presents a feasible and cost-efficient alternative for municipal waste management, contributing to sustainable construction practices. Lastly, Nodehi and Taghvaee [100] assessed the incorporation of WGP within the circular economy framework, demonstrating that WGP-based concrete supports innovative waste management strategies. Their research highlighted the contribution of WGP to sustainable construction practices by facilitating closed-loop recycling processes and minimizing dependence on landfill disposal.

c. Cost effectiveness and resource efficiency

Incorporating waste glass powder into concrete offers economic and environmental benefits that extend beyond sustainability, thereby enriching both economic and material efficiency. Because cement production is highly energy-intensive, partially replacing cement with WGP contributes to reduced manufacturing costs and a significant decrease in carbon emissions. Zhao et al. [101] conducted an extensive economic assessment of WGP in concrete-filled steel tubes, concluding that the partial replacement of cement with WGP improves economic feasibility by reducing raw material costs and prolonging service life through optimized durability. Similarly, Hassani et al. [102] reviewed green concrete incorporating glass powder and demonstrated its economic viability and global accessibility as an alternative to conventional cement. The analysis outlined the reduction of dependence on virgin materials while maintaining comparable mechanical performance by incorporating glass powder. Qayyum et al. [103] evaluated the social and economic feasibility of WGP, demonstrating that its application in the concrete industry reduces costs and facilitates further integration of waste in the construction industry. Furthermore, Manthos et al. [104] incorporated a life cycle assessment (LCA) methodology to waste glass geopolymer concrete, proving that substituting Portland cement for WGP lowers energy expenditure and construction costs. Finally, Abderraouf Belkadi et al. [105] conducted primary research on the two competing strategies of adding recycled concrete and WGP to cement mortars. They concluded that this approach increases the economic viability of the mortars and maintains their financial and structural performance. Although WGP is derived from post-consumer or industrial glass waste, its economic feasibility depends significantly on factors like collection, grinding, and transportation costs. In regions with limited access to glass waste processing facilities, the added energy and logistics expenses may offset the anticipated savings. However, studies suggest that when sourced and processed locally, WGP integration can still result in competitive cost–performance benefits. Future studies should incorporate comprehensive life cycle and cost–benefit analyses to support the practical implementation of this approach at scale.

d. Environmental and Economic Advantages of WGP

Compared to traditional SCMs, such as fly ash, GGBS, and silica fume, WGP exhibits competitive pozzolanic activity, particularly when processed to ultrafine sizes below 75 μm, as supported by multiple studies reviewed in Section 4.1. The fineness of WGP plays a crucial role in enhancing its reactivity by increasing the surface area, which contributes to improved compressive strength and reduced calcium hydroxide content in concrete. While fly ash and slag have long been used in concrete as SCMs, their availability is increasingly region-specific due to a decline in coal-based power and traditional steel production, as briefly discussed in Table 2. On the other hand, glass waste continues to be generated in large quantities from post-consumer sources and the packaging industry, making it a widely available and underutilized resource, as highlighted in the Introduction. Although silica fume is recognized for its superior pozzolanic reactivity due to its ultrafine particle size and high silica content, it is comparatively more expensive and less accessible, limiting its practical use in all regions. WGP, in contrast, offers both environmental and cost advantages by reducing landfill waste and enabling the partial replacement of cement, up to 30% in some cases, while maintaining acceptable mechanical performance. However, its effectiveness is highly sensitive to factors like particle size distribution, replacement level, and curing duration, which are consistently emphasized across the reviewed literature. This comparative understanding underscores WGP’s potential as a competitive and sustainable SCM, particularly in regions where glass waste is abundant and local grinding infrastructure exists.

## 6. Challenges and Future Research Directions

Implementing WGP usage in concrete significantly enhances mechanical performance and stability. However, its wide application is limited by pending issues requiring further research and suitable modification. As outlined in Table 3, the areas of concern that still require investigation include the alkali–silica reaction, limitations in workability, uncertainties associated with long-term durability, and thorough environmental impact assessments. Developing a complete understanding of these constraints is essential for advancing the feasibility of WGP in sustainable construction strategies.

The entire field can determine the real integration of WGP in concrete by addressing the above challenges through field and experimental research, thereby confirming that its presentation aligns with the structural and durability standards required for sustainable construction.

## 7. Conclusions

The waste integration of glass powder in concrete provides a viable approach to increasing significant features, such as durability, mechanical properties, and stability, in construction. The effect of filler and pozzolanic reactivity of waste glass powder tends to improve flexural, tensile, and compressive strength. However, optimal replacement levels must be maintained to prevent a decrease in strength.

- The incorporation of waste glass powder increases the durability of concrete by increasing its resistance to freeze–thaw cycles, sulfate attack, and chloride ion penetration. Moreover, it enhances the structural integrity of the concrete by significantly minimizing its permeability.
- The essential implementation of additional mitigation strategies, along with effective mix design, contributes to the prolonged quality performance of concrete structures by managing ASR expansion.
- The fine particles of waste glass powder affect the rheological properties and the water demand, ensuring necessary optimized mix proportions and the utilization of chemical admixtures to enhance workability.
- The inclusion of waste glass powder aids in the optimal recycling of glass waste, aligning with global sustainability objectives by suppressing dependency on cement and mitigating excessive carbon emissions.
- The absence of benchmarking and mass field studies stunts widespread implementation, outlining the need for progressive research and policy development.
- To substantiate the efficacy of WGP under diverse environmental conditions, several measures are essential, including conducting additional long-term studies, integrating hybrid materials, and conducting extensive durability assessments.

WGP can be systematically incorporated into sustainable construction practices, contributing to environmental impact reduction while also conserving structural integrity and long-term durability by mitigating these challenges. This review not only consolidates existing findings on the use of WGP in concrete but also presents a bibliometric trend analysis and a systematic comparison of mechanical, durability, and sustainability parameters across the literature. Unlike prior reviews, this work aligns WGP performance with SDGs, offers quantitative comparisons from diverse studies, and highlights gaps, such as ASR mitigation, long-term field validation, and economic barriers. These insights provide a roadmap for future research and policy, reinforcing WGP’s potential as a viable supplementary cementitious material in sustainable construction.

## Figures and Tables

**Table 1:** Summary of key studies on the chemical and physical properties of WGP in cementitious applications.

Authors	Key Findings	Chemical Composition	Physical Properties
Abellan-Garcia et al. (2024) [30]	WGP (28MRG and 7MRG) enhances UHPC microstructure and reduces porosity due to fine particle size.	SiO_2_ ~72–75%, Na_2_O > 10%	Jet-milled, 28 µm and 7 µm particle sizes; SEM showed smooth angular morphology.
Singh and Mohanty (2024) [31]	WGP in alkali-activated concrete (AAC) promotes C-S-H and C-(N)-A-S-H gel formation, refining microstructure.	SiO_2_ ~70.2%, Na_2_O ~11.4%	Median particle size 11.74 µm; specific surface area 6230 cm^2^/g; irregular particle morphology.
Yan et al. (2024) [47]	A 20% WGP replacement in UHPC increased compressive strength by 8.6% at 28 days; however, it also reduced the overall strength of the material.	SiO_2_ ~65.77%, CaO ~13.23%, Na_2_O ~9.50%	Density 2.69 g/cm^3^; significant surface area 1375.7 m^2^/kg; smooth irregular morphology.
Saribiyik et al. (2013) [48]	WGP (10–47%) in polymer concrete enhanced compressive strength (29%) and flexural strength (78%).	SiO_2_ ~72%	Fine particle size (<1 mm); improved compactness and filling capacity in polymer concrete.

**Table 2:** Influence of WGP on mechanical strength properties of concrete.

Authors	WGP Replacement (%)	Strength Parameter	Optimal Strength Improvement	Notable Findings
Lai and Chen (2024) [51]	15% GMP + 30% CW	Compressive	56.8% increase	Improved void filling and hydration
Khan et al. (2023) [52]	10% (cement) + 15% (sand)	Compressive	Highest CS	Excessive WGP reduces reactivity
Hitesh Kumar et al. (2021) [61]	9% WGP + 1% RSF	Tensile and Flexural	Maximum strength	Crack bridging and energy absorption
Orouji and Najaf (2023) [62]	10% WGP + 1.0% PP fibers	Flexural	Increased ductility	GFRP rebars increased brittleness
Mustafa et al. (2023) [64]	10% GP + 1% SF	Flexural	38.2% increase	Steel fibers improved crack resistance

**Table 3:** Key challenges and future research directions for WGP in concrete.

Challenge	Description	Future Research Directions
Alkali–Silica Reaction (ASR)	WGP has a high silica content, which can react with alkalis in cement, potentially leading to ASR-induced expansion and cracking.	Investigating chemical admixtures, nano-silica, or blended cement to reduce ASR effects.
Workability Issues	Fine WGP particles can alter the rheological properties of concrete, affecting flowability and requiring additional water or superplasticizers.	Developing advanced mix designs incorporating superplasticizers to improve workability.
Durability Concerns	While WGP enhances durability in some cases, long-term performance under harsh environmental conditions (e.g., marine exposure and high sulfate environments) requires further investigation.	Extensive field trials have been conducted over decades to evaluate the performance of WGP-based concrete.
Strength Limitations at High Replacement Levels	Excessive WGP replacement can reduce mechanical performance due to dilution effects and lower cementitious content.	Exploring hybrid approaches with fiber reinforcement or supplementary binders to counteract strength reductions.
Standardization and Field Applications	The absence of standardized guidelines for WGP usage in structural applications poses a challenge for widespread adoption.	Establishing global standards for WGP usage to facilitate adoption in structural applications.

## Data Availability

No new data were created or analyzed in this study. Data sharing is not applicable to this article.
